# Photodynamic Therapy for Facial Rejuvenation Combined Amber LED and Infrared Laser Irradiation: A Pilot Study Comparing ALA and MAL

**DOI:** 10.1002/jbio.70335

**Published:** 2026-08-10

**Authors:** Tassia Joi Martins, Juliana Teixeira Pedroso, Bruno Henrique Godoi, Juliana Guerra Pinto, Juliana Ferreira‐Strixino

**Affiliations:** ^1^ Laboratório de Fotobiologia Aplicada à Saúde (PhotoBioS), Instituto de Pesquisa e Desenvolvimento Universidade do Vale do Paraíba (UNIVAP) São José dos Campos São Paulo Brazil

**Keywords:** ALA, MAL, photodynamic therapy, rejuvenation, skin

## Abstract

The skin performs essential functions between the internal and external environments of the human body, such as protection against microorganisms, substances and radiation, maintenance of body temperature, prevention of excessive water loss, and production of vitamin D. However, many of these functions are reduced with aging and can be accentuated in photoaged skin. Photodynamic therapy (PDT) is a noninvasive technique used in the treatment of cancer, microbial infections, precancerous changes and for cosmetic purposes. Research on topical PDT suggests antibacterial, anti‐inflammatory and immunomodulatory effects on keratinocytes, fibroblasts, sebaceous glands and hair follicles. Therefore, this pilot study evaluated PDT on the face using 5‐aminolevulinic acid (ALA) and methyl 5‐aminolevulinate (MAL), combined with amber LED and laser irradiation in women aged 40–55 years. PDT was effective in rejuvenating photoaged skin, reducing fine lines, smoothing wrinkles, and improving skin softness, firmness and sagging, with minimal side effects.

## Graphical abstract

Topical ALA‐ and MAL‐based photodynamic protocols combined with amber light and infrared irradiation produced distinct PpIX fluorescence profiles in photoaged skin, revealing different photodynamic responses and supporting their potential use as complementary strategies for skin rejuvenation and improvement of photoaging‐related strategies.

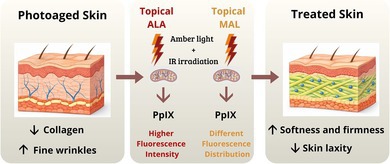

## 1 Introduction

In recent years, the growing interest in preserving skin appearance has stimulated the development of new therapeutic approaches and technologies [1, 2]. The concern with skin rejuvenation is not only related to esthetic demands but also to the prevention of skin diseases, since these treatments can also delay certain skin‐related dysfunctions [3, 4]. Indeed, it has already been described that cumulative skin damage can modify physiological patterns, such as reducing resistance to thermal and mechanical stress, impairing wound healing, and increasing the tendency to infections and neoplasms [5].

To delay cutaneous alterations associated with photoaging, several topical treatments can be employed, particularly retinoic acid and CO_2_ laser, which are considered the most effective. However, as a side effect, these treatments can cause redness, irritation in sensitive skin, pain, risk of hypopigmentation, and prolonged healing [6, 7]. In this scenario, the use of photodynamic therapy (PDT) is advantageous because it is less invasive and painless.

PDT consists of the application of a photosensitizer (PS) followed by irradiation at a specific wavelength. This combination produces reactive oxygen species (ROS) that can trigger a series of photochemical and biological responses in the skin [8]. Rejuvenation protocols using PDT can activate cellular repair mechanisms, including the upregulation of growth factors, stimulation of fibroblast activity, and remodeling of the extracellular matrix. These processes can lead to collagen I synthesis [9], the breakdown of extracellular membrane disorganized fibers, and enhanced epidermal turnover [10].

One of the advantages of PDT is the possibility of many adjustable parameters to optimize therapeutic outcomes. Variables such as the type of light source, PS, incubation time, energy density, number of sessions, and irradiation wavelength directly influence treatment efficacy. Wavelength selection plays a critical role due to differences in tissue penetration and biological effects.

Blue light is mainly associated with antimicrobial and anti‐inflammatory properties and has been widely applied in dermatological conditions such as psoriasis, rosacea, and dermatitis [11, 12]. Yellow light has demonstrated benefits in photoaging and melasma management, due to its ability to modulate melanogenesis [13]. Red light, owing to its deeper penetration, has been associated with improvements in skin elasticity and wrinkle reduction, likely related to oxidative stress modulation [12]. Near‐infrared (NIR) light has also been investigated for photoaging treatment, promoting cell proliferation through controlled ROS‐mediated signaling [13].

Combination of light wavelengths is one of the innovative approaches to photoaging. Yi et al. demonstrated an improvement in skin reducing wrinkles, pores, and texture using yellow light combined with red light and infrared LED [14].

However, combining wavelengths alone does not define a PDT protocol, since the photobiological response is mediated by the PS and its light absorption. Thus, understanding the properties of the PS is essential when designing multi‐wavelength approaches. Some studies have been investigating PS's in the dermatologic field for rejuvenation, such as 5‐aminolevulinic acid (5‐ALA) and its derivative, methyl aminolevulinate (MAL) [15, 16, 17]. Both compounds act as precursors of protoporphyrin IX (PpIX) in the heme biosynthetic pathway, leading to its intracellular accumulation and subsequent ROS generation upon irradiation. MAL has the advantage of being more lipophilic and could penetrate deeper into the skin [18].

Although these PS have been investigated for skin rejuvenation, studies integrating distinct spectral ranges within a single protocol remain limited. The combined use of amber LED irradiation, targeting PpIX absorption bands, and infrared laser exposure in the same treatment design has not yet been evaluated. Therefore, the present study aims to evaluate a novel PDT‐based protocol employing ALA and MAL, optimizing incubation time for each PS and investigating the effects of sequential amber and infrared irradiation. This strategy is intended to explore the potential synergistic interaction between PpIX‐mediated photochemical activation and infrared‐induced cellular signaling, contributing to improved strategies for photoaging.

## 2 Methods

### 2.1 Patient Selection

Prior to study initiation, the protocol was approved by the Research Ethics Committee of Vale do Paraíba University (CEP Univap, No. 5.942.007; March 14, 2023). All participants provided written informed consent before enrollment.

Participants were recruited through flyers on the Urbanova campus of the University of Vale do Paraíba, social networks, and the local community. Eleven participants were enrolled and randomly allocated through simple lottery‐based randomization into two groups: ALA‐PDT (*n* = 6) and MAL‐PDT (*n* = 5). This pilot study was conducted as a single‐center, prospective, and comparative study to assess the efficacy of the combined use of amber LED and infrared laser light, in addition to comparing ALA and MAL. The inclusion and exclusion criteria are presented in Table 1.

**TABLE 1:** Inclusion and exclusion criteria for this study.

Inclusion criteria	Exclusion criteria
Female aged 40–55 years	History of porphyria, photosensitivity
Fitzpatrick skin phototypes I–IV	Recent use of photosensitizing drugs (including systemic retinoids or topical retinoic acid)
Availability to attend all evaluation and treatment sessions	Recent skin disease, surgery, trauma, systemic illness affecting skin condition
Clinically diagnosed facial photoaging characterized by irregular hyperpigmentation, textural alterations	Uncontrolled systemic diseases (e.g., diabetes, hypertension, autoimmune disorders)
Presence of fine and deep wrinkles	Cosmetic procedures (botulinum toxin, dermal fillers, laser resurfacing, chemical peels, dermabrasion, or non‐ablative rejuvenation) within 6 months before enrollment
	Pregnancy or breastfeeding
	Impaired wound healing on clinical examination
	Refusal to authorize the use of data and images for research purposes

Standardized facial photographs were obtained with anonymization procedures, including placement of a black bar over the periocular region to preserve participant identity.

### 2.2 Procedures and Clinical Protocol

#### 2.2.1 Clinical Assessment

Skin hydration and oiliness parameters were quantified as percentages using the Skin Analyzer Digital SKN1501. Skin phototype was determined according to the Fitzpatrick Classification [19] using the Digital Skin Phototype Analyzer SKN1802 (SkinUp).

Anamnesis and structured questionnaires collected information regarding lifestyle habits, sun exposure and photoprotection practices, water intake, prior esthetic procedures and self‐perceived skin concerns. Following completion of the treatment protocol, participants completed a standardized self‐assessment questionnaire regarding their experience with PDT.

#### 2.2.2 PDT Protocol

The treatment area was cleansed with gel cleanser, exfoliant, and micellar water for combination‐to‐oily skin, followed by antisepsis with 2% chlorhexidine.

Baseline skin fluorescence was assessed using the Lince detection system (MM Optics) prior to topical precursor application.

The PS precursor (2% formulation in cream base) was applied as a thin, uniform layer under light‐protected conditions. After the predetermined incubation period (90 min), PpIX formation was confirmed via fluorescence imaging, and pre‐irradiation images were recorded. Following light exposure, post‐treatment fluorescence images were obtained for PpIX quantification.

#### 2.2.3 Irradiation Parameters

Irradiation (Table 2) was performed using the Elite‐Olympus device (DMC Equipment) with a handpiece containing eight amber LEDs (590 ± 10 nm) and two infrared lasers (808 ± 10 nm), each with a maximum power of 100 mW. Light delivery consisted of five 1‐min phases: one continuous followed by four pulsed emissions (60, 120, 150, and 180 pulses/min).

**TABLE 2:** Estimated optical parameters of the combined amber LED and infrared laser photodynamic therapy protocol.

Light source	Wavelength (nm)	Number emitters	Emission mode	Nominal power emitter (mW)	Total power (mW)	Nominal treatment area (cm^2^)	Instantaneous irradiance (mW/cm^2^)	Irradiation time (s)	Estimated fluence (J/cm^2^)
Infrared laser	808 ± 10	2	Continuous	100	200	25	8	300	2.4
Amber LED	590 ± 10	8	Continuous (1 min) + pulsed (4 min)	100	800	25	32 (instantaneous ON)	137.5–315^a^	4.4–10.1^a^
Combined (IR + amber)	—	—	Simultaneous	—	—	25	—	—	6.8–12.5^a^

^a^Estimated fluence (J/cm²) was calculated as irradiance (W/cm²) × irradiation time (s).

Irradiance and fluence values were calculated based on the manufacturer's nominal output and the reported treatment area (25 cm^2^). Amber fluence was estimated considering the pulsed emission duty cycle. Because pulse width may vary (152–500 ms), amber fluence is reported as a range.

#### 2.2.4 Treatment Schedule and Follow‐Up

A total of four treatment sessions were performed at 30‐day intervals. Participants returned 30 days after the fourth session for follow‐up evaluation and standardized photographic documentation.

#### 2.2.5 Safety Monitoring

Adverse events were recorded at each session and included pain, burning sensation during precursor application or irradiation, transient photosensitivity, edema, erythema, desquamation, and post‐inflammatory pigmentation changes. Supportive measures such as cold compresses and soothing topical formulations were permitted when necessary.

### 2.3 Image Acquisition and Analysis

Image acquisition was standardized throughout the study using the smartphone camera (Apple iPhone SE 2020, 12‐megapixel): photographs were taken with a standard distance between the camera and the patient. To ensure this, a tripod and a table were used, maintaining a consistent distance of approximately 15 cm. Room lighting was also standardized, and all images were captured with the flash enabled.

Fluorescence images were acquired using the Lince detection system (MM Optics) to capture PpIX emission. In this system, a violet light (405 ± 10 nm) is directed applied onto the tissue, and the emitted response is filtered ensuring that only the light emitted by the tissue is visualized. The quantitative fluorescence intensity analysis was performed using ImageJ software (NIH, USA). Images were converted into RGB channels, and the red channel was selected for fluorescence quantification due to the emission characteristics of PpIX. A consistent threshold value was applied across all images to minimize analytical bias. Mean fluorescence intensity values were extracted for each ROI. Fluorescence measurements were normalized to baseline values (prior to any treatment) and expressed as fold change relative to control. Data distribution was assessed prior to inferential analysis. Ten predefined anatomical regions of interest (ROIs) were analyzed: (1) central forehead; (2) left forehead; (3) left orbicularis oculi; (4) left malar region; (5) left nasolabial fold; (6) mentum; (7) right nasolabial fold; (8) right malar region; (9) right orbicularis oculi; and (10) right forehead.

For winkles measurements, specific facial ROIs were defined, including the forehead, nasolabial fold, orbicularis (front part), malar, and lateral periorbital. These areas were selected based on the presence of visible static wrinkles. The same ROIs were consistently analyzed in both baseline and post‐treatment images. All images were acquired under standardized conditions, including controlled distance, lighting, and subject positioning, to ensure reproducibility. This methodology provides a semi‐quantitative assessment, enabling relative comparisons between baseline and post‐treatment conditions rather than absolute measurements. Images were converted to 8‐bit grayscale, and wrinkle length was determined using the straight‐line tool in ImageJ. Results were expressed as percentage variation relative to baseline values.

### 2.4 Statistical Analysis

Data distribution was assessed for normality using the Shapiro–Wilk test. As all datasets met the assumption of normality (*p* > 0.05), parametric analysis was applied. Comparisons between groups were performed using Student's *t*‐test (two‐tailed). Exact *p* values for all comparisons are provided in Table S1. In addition, confidence intervals (95%) were calculated to estimate the precision of the observed effects. Differences were considered statistically significant when *p* < 0.05.

## 3 Results

### 3.1 Questionnaire Analysis

Before the treatment, all participants completed a questionnaire with their perceived facial condition, which highlighted their main complaints: fine wrinkles, static wrinkles, dryness, hyperpigmentation, sagging, periorbital puffiness, and periorbital hyperpigmentation.

Regarding facial skin care, six participants use sunscreen daily, four use hydration cosmetics, and one participant uses vitamin C. Some esthetic procedures performed before this clinical trial included microneedling, peeling, laser (at least 6 months earlier), botulinum toxin injections, thread lifting, and collagen biostimulators (about 2 years earlier).

About general habits, 1 participant is a smoker, 10 didn't use any contraceptive methods, and 6 are in menopause. Regarding daily water intake, 2 participants reported drinking more than 10 glasses of water, 4 participants reported drinking 7–9 glasses, and 5 participants reported drinking 6 glasses or fewer. Among participants with lower hydration, skin dryness can be observed.

For current sun exposure and protection, nine participants report less than 1 h per day, one participant between 1 and 3 h, and one participant between 3 and 6 h. For all participants, sun exposure occurs during habitual commuting. Only six participants use sunscreen daily (even on cloudy days), but only three reapply it.

To understand lifestyle habits, the participants were questioned about sun exposure up to the age of 15: five participants were exposed to the sun for 1–3 h per day, four participants for 3–6 h, and two participants for less than 1 h, while sunscreen was never used by four participants and rarely used by 6. Between 26 and 35 years of age, most participants were exposed for less than 1 h per day (8), but only two always used sunscreen, and the majority (5) used it rarely.

Approximately 30 days after the last session, participants completed the post‐treatment self‐assessment form. Most participants (8) rated discomfort during the sessions as minimal, and none rated it as maximal. Only one participant (in the ALA group) did not notice any skin improvement and would not recommend the treatment. All others would recommend it, highlighting improvements in fine lines (most frequently; five participants), radiance (3), oiliness, spot reduction, and dryness. The mean satisfaction score was 8.3 out of 10, with the ALA group achieving the highest ratings. During the treatment months, nine participants reported using sunscreen more frequently, four increased daily water intake, and two reduced body weight.

### 3.2 Evaluation of Facial Images, Hydration, and Skin Oil Levels

Prior to the treatments, the participants' skin phototypes were evaluated, and all were identified as Fitzpatrick phototype IV.

**FIGURE 1:**
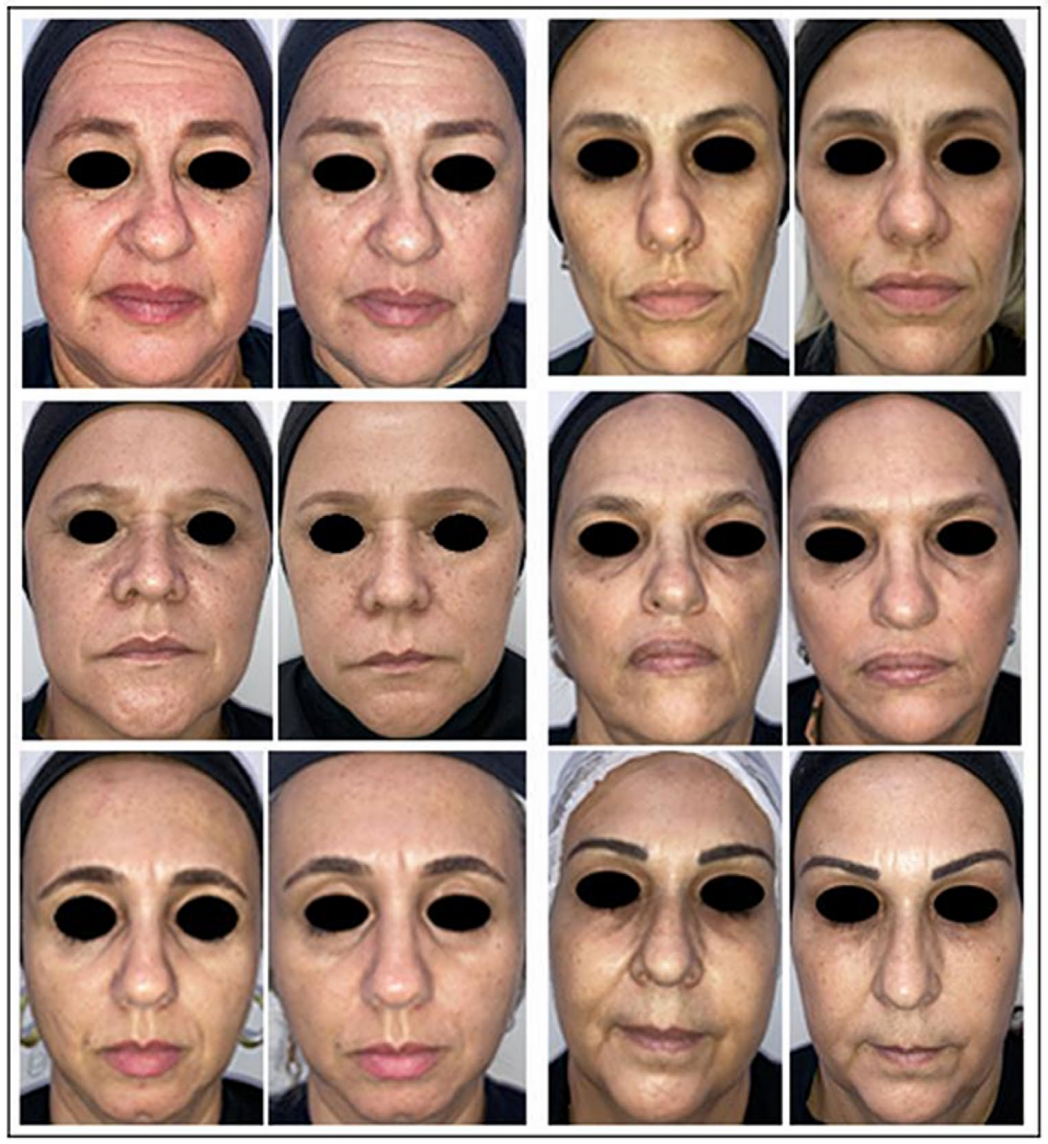
Photographic documentation of participants in the ALA‐PDT group. The images show, respectively, the baseline (control) condition and approximately 30 days after the fourth treatment session.

**FIGURE 2:**
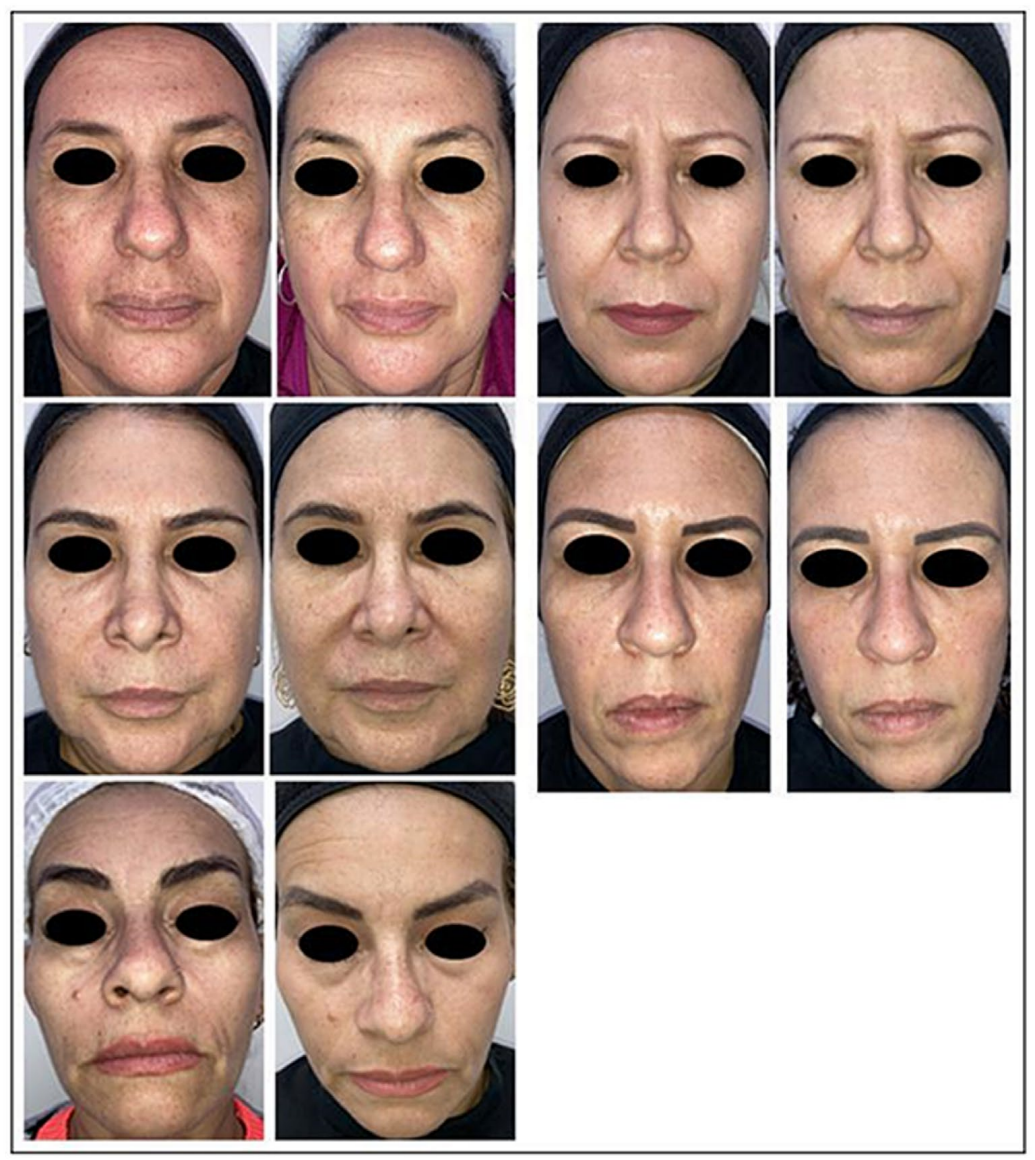
Photographic documentation of participants in the MAL‐PDT group. The images show, respectively, the baseline (control) condition and approximately 30 days after the fourth treatment session.

Qualitative treatment analysis was performed by photographic documentation before the first evaluation session and prior to each PDT session. Treatment efficacy was observed for both groups (ALA‐PDT and MAL‐PDT treatment), particularly through the reduction of fine wrinkles in the eye region, facial contour, and attenuation in nasolabial fold prominence (Figures 1 and 2).

Skin hydration and oiliness were evaluated prior to each session in four facial regions (forehead, right and left malar areas, and chin). Both parameters showed no significant changes, as the measurements exhibited considerable variability (Figures S1 and S2).

### 3.3 Quantitative Analysis: Fluorescence Quantification and Wrinkles Measurements

For a more detailed evaluation of the proposed protocol, wrinkles were assessed for length, and the data were converted to percent change. The variation in the ALA group ranged from 3% to 19%, with a marked decrease in the nasolabial and malar folds. In the MAL group, a length decrease was observed in four of five measured areas, with variation ranging from 11% to 25% (Table 3).

**TABLE 3:** Wrinkle length mean percent change of treated patients. The arrows value indicates a decrease (↓) or an increase (↑) in wrinkle length.

Analyzed areas	ALA group (*n* = 6), mean ± SD (%)	MAL group (*n* = 5), mean ± SD (%)
Forehead	↑6.30 **±** 27.81	↓13.5 **±** 25.76
Nasolabial fold	↓11.7 **±** 27.84	↑13.8 **±** 16.89
Orbicularis (front part)	↑3.40 **±** 8.70	↓9.68 **±** 7.41
Malar	↓7.07 **±** 8.31	↓25.3 **±** 4.52
Lateral periorbital	↑19.1 **±** 39.95	↓11.0 **±** 37.54

**FIGURE 3:**
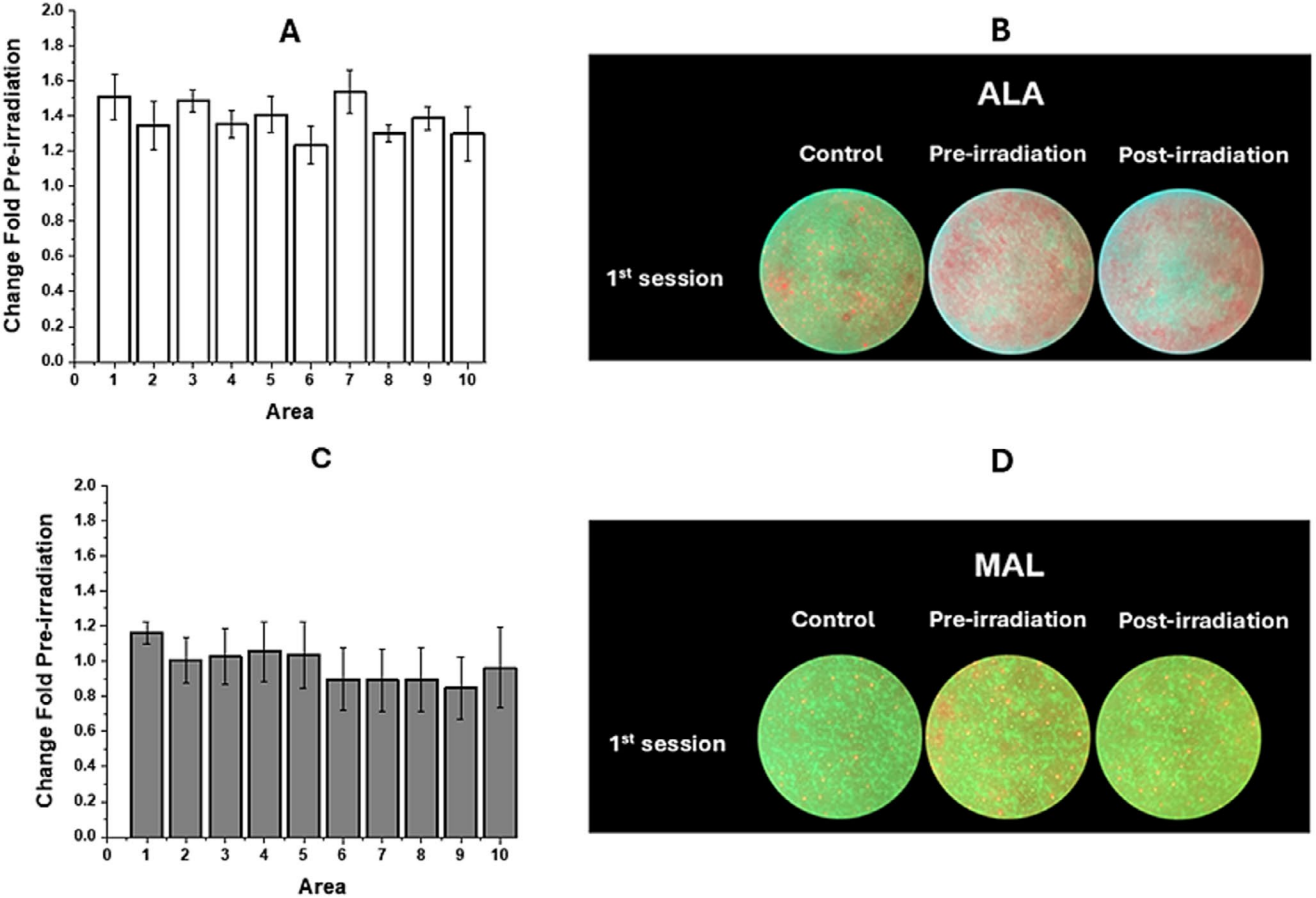
Fluorescence emission observed due to PpIX production by the ALA and MAL compounds during the first session: (A) ratio between the fluorescence observed prior to irradiation and control for (change fold), ALA compound; (B) fluorescence observed in patient skin treated with ALA; (C) ratio between the fluorescence observed prior to irradiation and control for (change fold), MAL compound; and (D) fluorescence observed in patient skin treated with MAL.

Fluorescence quantification was also performed in all patients to monitor PpIX levels before and after PDT (Figure 3). The fluorescence was normalized as a fold change relative to the control (before the application of the PSs).

For group ALA‐PDT, in the first session, fold change ranged from 1.2 to 1.5 across all areas studied (Figure 3A,B), suggesting a fluorescence increase before application in comparison with control. The same behavior was observed after irradiation of the same analyzed areas.

When comparing the ALA‐PS application before and after irradiation, no statistical significance was observed for the seven evaluated regions, since fold change remained close to unity. The fourth session reproduced the same pattern observed in the first session.

In the MAL‐PDT group, no significant difference in fluorescence was detected immediately after compound application compared to control and after irradiation (Figure 3C,D).

Regarding intersession comparisons, a significant decrease in fluorescence across sessions was observed in the ALA‐PDT group, and no decrease was observed in the MAL‐PDT group. Direct comparison between PSs during the first session revealed significantly higher fluorescence values for ALA than for MAL across all evaluated regions. This pattern persisted after irradiation. Detailed statistical analyses, including fold changes and exact *p* values for all comparisons, are provided in Tables S1 and S2.

## 4 Discussion

PDT using ALA and MAL resulted in qualitatively satisfactory skin rejuvenation outcomes, as reported by the patients in this study. Considering the differences observed between the two treatments, all ALA‐group participants reported positive changes in their skin in response to the treatment. Among patients treated with MAL, one participant did not perceive any improvement and would not recommend the treatment. This participant was the only one to report being a smoker in the anamnesis. Smoking can induce unfavorable changes in the skin, such as loss of elasticity and accelerating natural aging, which may hinder the achievement of positive outcomes in rejuvenation therapies [20, 21].

The qualitative analysis and self‐perception performed with the participants indicate responses consistent with those described in the literature. A study using a 5% ALA formulation, with 30‐min incubation and 633 nm light irradiation for 20 min, showed a significant reduction in fine lines in the periorbital region during clinical evaluation in four of six participants. Skin smoothness was also improved in all treated individuals [22]. Ruiz‐Rodriguez and colleagues reported that the combination of MAL and red light could induce moderate improvements in fine lines, tactile roughness, and skin firmness [23].

Regarding quantitative analysis, no significant difference in the percent change in wrinkle length was observed between groups, suggesting that the patient's perceived improvement may be mainly associated with fine wrinkles rather than static or deep wrinkles. Indeed, several studies support these findings, reporting the use of both ALA and MAL in PDT for the treatment of fine lines [16, 24, 25]. Szeimies et al. have also emphasized that PDT is not the preferred option for deep wrinkles, for which other therapeutic approaches are more appropriate [26].

In PDT, treatment efficacy depends on the PS and its associated parameters. Incubation time is one of the main variables in ALA‐PDT and MAL‐PDT protocols reported in the literature, typically ranging from 1 to 20 h [27, 28]. In our study, a prior evaluation established an incubation period of 90 min, which was found to be sufficient to ensure the occurrence of the essential photochemical reactions of the therapy. Incubation and application times are important parameters, as they can influence PpIX production.

PpIX production through MAL and ALA requires a biosynthetic pathway that involves a series of enzyme‐mediated reactions [29, 30]. For MAL, some studies have reported differences in PpIX formation compared to ALA, particularly regarding the rate and extent of PS accumulation [31, 32]. These findings are consistent with the present fluorescence analyses, which indicate variations in PpIX production depending on the precursor and incubation time. Considering these differences in PpIX formation profiles, Fujita and colleagues [33] reported that a 50% ALA‐MAL mixture in treatment increases PpIX production in terms of quantity, homogeneity, and duration compared to ALA or MAL alone, positively impacting photodynamic damage and optimizing PDT.

Furthermore, for the ALA‐PDT group, the fluorescence intensity decreased over the treatment sessions. This observation is consistent with previous reports and is explained by the photobleaching of PpIX during irradiation. In addition to photochemical degradation, differences in fluorescence intensity may also reflect variations in the skin penetration of PS across patients. Juzeniene and coworkers [34] reported that the penetration of these PS's could also be related to stratum corneum and epidermis thickness after sun exposure, since human skin frequently exposed to solar radiation produces less PpIX. The same behavior was not observed for the MAL‐PDT group. The extent of photobleaching depends on factors such as fluence, irradiance, local oxygen availability, and tissue microenvironment, which may have limited the observable decrease in fluorescence under the present conditions.

Comparing the two treatments, it was observed that ALA‐PDT showed higher fluorescence intensity in the first and fourth sessions. Lesar and coworkers [35] reported a correlation between fluorescence intensity and application time, suggesting that longer MAL application times favored the PpIX production. This factor aligns with our results showing that ALA‐PDT showed higher PpIX fluorescence intensity when applied for shorter times.

The effectiveness of PDT is also influenced by the selection of the light source and the dosimetry. An association between amber LED light and infrared laser was employed once and acts on tissue repair, modulates inflammatory responses, and skin vascularization [36]. The infrared laser provides analgesic effects, enhances hydration across different skin layers, and improves muscle tone, and the combination of both reduces skin flaccidity and promotes a facial lifting effect.

The irradiation mode combining LED and laser—with the first minute delivered in continuous emission and the following four minutes in pulsed emission—was designed to ensure efficient, comfortable light delivery, as prolonged continuous exposure can cause tissue heating. Yuzhakova et al. [37] reported that continuous and pulsed irradiation modes may induce distinct biological mechanisms: pulsed modes can result in lower singlet oxygen production, leading to apoptosis rather than necrosis in tumor tissues, reoxygenation during the dark periods, and may provide deeper tissue penetration. Continuous irradiation may induce a beneficial thermal effect by promoting vasodilation and increasing blood flow.

Overall, among the parameters tested in this study, PDT with ALA and MAL, associated with an amber LED and an infrared laser, showed indications of beneficial effects on rejuvenating photoaged skin, particularly in fine wrinkles and firmness. The ALA‐treated group tended to exhibit an improvement compared to the MAL group, as reflected in mainly patient‐reported observations.

## 5 Conclusion

The application of PDT using ALA and MAL was associated with improvements in facial photoaging skin, demonstrating reductions in fine lines, skin softness, firmness, and decreased skin laxity. Among the PS, ALA showed a slightly pronounced rejuvenation effect, probably due to the shorter application time used in this protocol. The 2% concentration of PS enabled these outcomes with minimal side effects. Moreover, the interaction of PpIX, produced after 90 min of incubation, with the combined amber and infrared wavelengths contributed to the observed rejuvenating effects.

The analysis of these results underscores the importance of personalized treatment in the rejuvenation area. The optimization of protocols for different skin phototypes and specific skin characteristics is a prominent area of interest, underscoring the need for future studies to customize therapy and improve both clinical efficacy and patient comfort.

## 6 Limitations and Future Perspectives of the Study

In this study, we investigated a novel rejuvenation protocol combining amber LED and infrared laser irradiation with two well‐established PS in PDT. The results demonstrated improvements in fine wrinkles and skin firmness in middle‐aged women. However, no notable effects were observed on deeper, static wrinkles at this stage. Future work will focus on optimizing the protocol to determine whether this combination of light sources can also elicit measurable improvements in more profound dermal alterations.

As a pilot study, this work has limited statistical power due to the small sample size. However, the findings provide preliminary insights and should be further investigated in larger populations. In addition, control groups will be included (e.g., light‐only and PS‐only) to enable a more comprehensive assessment of the individual contribution of each component. We also suggested investigating the same protocol for longer application times to evaluate if MAL‐PDT treatment could be improved with the same light source used in this protocol.

## Author Contributions

**Tassia Joi Martins:** analysis and data interpretation, writing – original draft, review. **Juliana Teixeira Pedroso:** data acquisition, methodology, writing – original draft. **Bruno Henrique Godoi:** data curation, formal analysis, writing. **Juliana Guerra Pinto:** conceptualization, formal analysis, visualization, writing, review, and editing. **Juliana Ferreira‐Strixino:** conceptualization, formal analysis, funding acquisition, supervision, visualization, review, and editing.

## Funding

This work was supported by Coordination for the Improvement of Higher Education Personnel (CAPES), Brazil, Financing Code 001 and National Council for Scientific and Technological Development (CNPq) Process No. 315037/2025‐3.

## Conflicts of Interest

The authors declare no conflicts of interest.

## Supporting information


**Figure S1:** Oilness percentage parameter for the participants treated with ALA and MAL.
**Figure S2:** Skin hydration percentage parameter for the participants treated with ALA and MAL.
**Table S1:** Summary of fluorescence intensity comparisons in ALA‐PDT and MAL‐PDT groups. Fold changes and corresponding *p* values are presented for comparisons performed for each photosensitizer (before vs. after irradiation, between sessions) and between photosensitizers (ALA vs. MAL). Statistical significance was defined as *p* < 0.05. *ns = no significant.
**Table S2:** Statistical comparison between ALA‐PDT and MAL‐PDT groups for wrinkles measures.

## Data Availability

The data that support the findings of this study are available in the Supporting Information of this article.
