# Supplementary material for: Photodynamic Therapy for Facial Rejuvenation Combined Amber LED and Infrared Laser Irradiation: A Pilot Study Comparing ALA and MAL
Source: J Biophotonics. 2026 Aug 10;19(8):e70335. doi: 10.1002/jbio.70335 (PMC13456946; doi:10.1002/jbio.70335)
Supplement: Supplementary file 1 [file JBIO-19-e70335-s001.docx]

**Photodynamic Therapy for Facial Rejuvenation combined amber LED and infrared laser irradiation: A Pilot Study comparing ALA and MAL**

Tassia Joi Martins^1^, Juliana Teixeira Pedroso^1^, Bruno Henrique Godoi ^1^, Juliana Guerra Pinto^1^, Juliana Ferreira-Strixino^1*^.

^1^Laboratório de Fotobiologia Aplicada à Saúde (PhotoBioS), Instituto de Pesquisa e Desenvolvimento, Universidade do Vale do Paraíba (UNIVAP). Avenida Shishima Hifumi, 2911, Urbanova, São José dos Campos, SP, CEP: 12244-000.

*corresponding author: [juferreira@univap.br](mailto:juferreira@univap.br)

**Supplementary Material**

**Table of Contents**

- **Figure S1…………………………………………………………..…………………..1**
- **Figure S2……………………………………………………………..………………..1**
- **Table S1………………………………………………………………………………..2**
- **Table S2………………………………………………………………………………..2**

**Figure S1**. Oilness percentage parameter for the participants treated with ALA (figure 1A) and MAL (figure 1B).

**Figure S2**. Skin hydration percentage parameter for the participants treated with ALA (figure 2A) and MAL (figure 2B).

**Table S1.** Summary of fluorescence intensity comparisons in ALA-PDT and MAL-PDT groups. Fold changes and corresponding p-values are presented for comparisons performed for each photosensitizer (before vs. after irradiation, between sessions) and between photosensitizers (ALA vs. MAL). Statistical significance was defined as p < 0.05. *ns = no significant.

| **Comparison** | **Group** | **Fold Change** | **Exact p-value** | **Interpretation** |
| --- | --- | --- | --- | --- |
| 1^st^ session vs control  (Session 1) | ALA-PDT | 1.3 to 1.5 | 0.00096 | Significant increase |
| After irradiation vs control  (Session 1) | ALA-PDT | 1.2 to 1.5 | 0.0019 | Significant increase |
| Before x after irradiation  (Session 1 and 4) | ALA-PDT | ~ 1 | 0.32 | ns* |
| 1^st^ session vs control  (Session 1) | MAL-PDT | 0.89-1.16 | 0.053 | Significant (no difference in the values) |
| After irradiation vs control  (Session 1) | MAL-PDT | ~ 1 | 0.38 | ns* |
| Before x after irradiation  (Session 1 and 4) | MAL-PDT | ~ 1 | 0.31 | ns* |
| ALA vs MAL (After Application – Session 1) | Both | — | 9.5 × 10⁻⁷ | ALA > MAL |
| ALA vs MAL (After Irradiation – Session 1) | Both | — | 5.7 × 10⁻⁷ | ALA > MAL |

**Table S2.** Statistical comparison between ALA-PDT and MAL-PDT groups for wrinkles measures.

| **Parameter** | **Value** |
| --- | --- |
| Test used | Unpaired t test (two-tailed) |
| Sample size (ALA *vs* MAL) | 6 *vs* 5 |
| p-value | 0.2181 |
| Significance (p < 0.05) | Not significant (ns) |
